# Postsplicing-Derived Full-Length Intron Circles in the Protozoan Parasite *Entamoeba histolytica*

**DOI:** 10.3389/fcimb.2018.00255

**Published:** 2018-08-03

**Authors:** María S. Mendoza-Figueroa, Eddy E. Alfonso-Maqueira, Cristina Vélez, Elisa I. Azuara-Liceaga, Selene Zárate, Nicolás Villegas-Sepúlveda, Odila Saucedo-Cárdenas, Jesús Valdés

**Affiliations:** ^1^Departamento de Bioquímica, Centro de Investigación y de Estudios Avanzados del Instituto Politécnico Nacional, Mexico City, Mexico; ^2^Departamento de Biología Celular, Centro de Investigación y de Estudios Avanzados del Instituto Politécnico Nacional, Mexico City, Mexico; ^3^Posgrado en Ciencias Genómicas, Universidad Autónoma de la Ciudad de Mexico, Mexico City, Mexico; ^4^Departamento de Biomedicina Molecular, Centro de Investigación y de Estudios Avanzados del Instituto Politécnico Nacional, Mexico City, Mexico; ^5^Departamento de Histología, Facultad de Medicina, Universidad Autónoma de Nuevo León, Monterrey, Mexico; ^6^División de Genética, Centro de Investigación Biomédica del Noreste, Instituto Mexicano del Seguro Social, Monterrey, Mexico

**Keywords:** ncRNA, circular RNA, Dbr1, virulence, splicing, 5′ss, branch point sequence

## Abstract

Noncoding circular RNAs are widespread in the tree of life. Particularly, intron-containing circular RNAs which apparently upregulate their parental gene expression. *Entamoeba histolytica*, the causative agent of dysentery and liver abscesses in humans, codes for several noncoding RNAs, including circular ribosomal RNAs, but no intron containing circular RNAs have been described to date. Divergent RT-PCR and diverse molecular approaches, allowed us to detect bona fide full-length intronic circular RNA (flicRNA) molecules. Self-splicing reactions, RNA polymerase II inhibition with Actinomycin D, and second step of splicing-inhibition with boric acid showed that the production of flicRX13 (one of the flicRNAs found in this work, and our test model) depends on mRNA synthesis and pre-mRNA processing instead of self-splicing. To explore the cues and factors involved in flicRX13 biogenesis *in vivo*, splicing assays were carried out in amoeba transformants where splicing factors and Dbr1 (intron lariat debranching enzyme 1) were silenced or overexpressed, or where Rabx13 wild-type and mutant 5′ss (splice site) and branch site minigene constructs were overexpressed. Whereas SF1 (splicing factor 1) is not involved, the U2 auxiliary splicing factor, Dbr1, and the GU-rich 5′ss are involved in postsplicing flicRX13 biogenesis, probably by Dbr1 stalling, in a similar fashion to the formation of ciRNAs (circular intronic RNAs), but with distinctive 5′-3′ss ligation points. Different from the reported functions of ciRNAs, the 5′ss GU-rich element of flicRX13 possibly interacts with transcription machinery to silence its own gene in *cis*. Furthermore, introns of *E. histolytica* virulence-related genes are also processed as flicRNAs.

## Introduction

Circular RNAs (circRNAs) are a group of noncoding RNAs (ncRNAs) that originated from protein coding genes. They were described nearly 40 years ago through electron microscopy studies (Hsu and Coca-Prados, 1979); however, their prevalence in different cellular conditions, biogenesis and possible functions have been explored only recently with the advent of massive sequencing of the transcriptomes.

Most of them still lack a specific function but it has been described that some may act as postranscriptional regulators in the cytoplasm or may act as cotranscriptional regulators in the nucleus (Hansen et al., 2013; Memczak et al., 2013; Ashwal-Fluss et al., 2014; Li et al., 2015), suggesting that circRNAs have important functions in modifying gene expression.

The best studied circRNAs are exon-coded cytoplasmic microRNA sponges (Hansen et al., 2013; Memczak et al., 2013), while others consist of exons that include full intronic sequences, named EIciRNAs, which compete with splicing to control their parental gene expression in *cis* via their interaction with RNA Pol II and the U1 small nuclear RNA at their respective promoter regions (Ashwal-Fluss et al., 2014; Li et al., 2015). The biogenesis of circRNAs and EIciRNAs involves backsplicing or is mediated by an exon-containing lariat precursor, pathways favored by complementary sequences, such as ALU repeats, in the introns bracketing the circularized exons and in some cases mediated by the splicing factors *muscleblind* or *quaking* (Jeck et al., 2013; Memczak et al., 2013; Ashwal-Fluss et al., 2014; Barrett et al., 2015; Conn et al., 2015; Ivanov et al., 2015).

Cytoplasmic (and some nuclear) stable intronic sequence RNAs (sisRNAs) and ciRNAs conform an additional type of splicing-related circular RNA molecules that escape debranching reaction of intron lariats (Zhang et al., 2013; Talhouarne and Gall, 2014). Cytoplasmic sisRNAs are lariats lacking the 3′ terminal tail (approximately 30 nt upstream the 3′ss) and are present during early embryogenesis in the cytoplasm of *Xenopus tropicalis*. It has been proposed that they avoid debranching before leaving the nucleus since they are opened by nuclear, but not cytoplasmic, extracts of *Xenopus laevis* (Talhouarne and Gall, 2014). Conversely, ciRNA production in human cells depends on the presence of a GU rich sequence element close to the 5′ss and a C-rich element near to the branch site (BP). These elements are not present in introns that are not circularized, and more importantly they enable the lariats that contain such elements to escape debranching. Therefore, Dbr1 appears to be involved in the biogenesis of sisRNAs and ciRNAs through particular mechanisms not yet described. The ciRNAs interact with the phosphorylated RNA Pol II to activate the transcription of its parental genes (Zhang et al., 2013).

To date, the best characterized circular RNA molecules have been described in multicellular eukaryotes, yeasts, *Plasmodium falciparum* and *Dictyostelium discoideum* (Wang et al., 2014), but not in other protists of clinical relevance, such as *Entamoeba histolytica*, the etiologic agent of amoebiasis which still causes over 55,000 annual deaths worldwide, and 2.2 million disability-adjusted life years (Lozano et al., 2012; Murray et al., 2012). However, *E. histolytica* is not devoid of regulatory ncRNAs which include stressed-induced self-circularized 5′-external transcribed spacer rRNAs (Gupta et al., 2012), and microRNAs (De et al., 2006; Mar-Aguilar et al., 2013).

*Entamoeba histolytica* possess small introns of ≈75 nt in length with extremely conserved 5′ and 3′ss, GUUUGUU and UAG, respectively (Wilihoeft et al., 2001; Davis et al., 2007; Lorenzi et al., 2010; Hon et al., 2013). The BP sequence (YNYYRAY) of amoebic introns has been identified only *in silico* and lacks the conservation maintained in other protozoans (Wilihoeft et al., 2001; Vanácová et al., 2005; Davis et al., 2007).

Here, we explored the existence of *E. histolytica* splicing products similar to the vertebrate circRNAs, EIcircRNAs, or ciRNAs. For the first time we report circular ncRNA molecules from different amoeba gene products, including some overexpressed in virulence. Different from their vertebrate counterparts, these are conformed by full-length intronic circular RNAs (flicRNAs). Characterization of the intron circle of *RabX13* (flicRX13) showed that it originates from transcription/splicing of its parental gene and is more stable than the IR (intron retained), spliced and lariat variants that originated thereof. FlicRX13 biogenesis can be attributed to the intronic 5′ss GU-rich element and possibly to Dbr1 activity, which we are still investigating. Due to their distinctive 5′-3′ss ligation points, we propose that flicRNAs might arise from stalled Dbr1 with the aid of additional factors. Different from the reported functions of ciRNA, minigene intron constructs carrying 5′ss GU-rich element mutants evidenced a *cis*-regulatory role of flicRX13 silencing its parental gene, possibly via interaction with factors of the transcription machinery.

## Materials and methods

### *E. histolytica* cultures and drug treatments

Trophozoites of the *E. histolytica* strain HM1:IMSS were axenically cultured in Trypticase-yeast extract-iron serum (TYI-S-33) medium at 37°C and harvested as described (Diamond et al., 1995). For *in vivo* assays (splicing, inhibition of second step of splicing or Dbr1 inhibition), 10^6^ log phase (48 h) wild type or transfectant trophozoites per experimental point, either alone or treated with 1.3 μM Actinomycin D (Sigma Aldrich) as described (López-Camarillo et al., 2003), or with different concentrations of boric acid (pH 7.9) (Shomron and Ast, 2003) during 1.5 h. After treatments, cells were harvested, and total RNA was isolated and analyzed by reverse transcription (RT) followed by polymerase chain reaction (PCR) amplification of the DNA.

### RNA and DNA isolation

Genomic DNA (gDNA) and total RNA were isolated using the TRIzol Reagent as specified by the manufacturer (Invitrogen). For RNA isolation, preparations were treated with RQ1 DNase (Promega) or with RNase R (Epicentre), when specified. For gDNA purification, preparations were treated with RNases T1 and A.

### Retro transcription and polymerase chain reactions

All primers and PCR conditions are listed in Table S1. *RabX13* (EHI_065790)*, U6 snRNA* (U43841)*, 18S rRNA* (AB426549)*, RNA Pol II* (EHI_087360), and *EhActin* (EHI_107290) gene expression were monitored by RT-PCR using specific primer pairs, M-MLV retro transcriptase, and Taq DNA Polymerase, as specified by the manufacturer (Invitrogen). All PCR products were cloned into the pCR2.1 plasmid vector (Invitrogen) and sequenced.

To detect circular RNA molecules, specific outward facing primer pairs targeted to the introns of the *E. histolytica* genes *RabX13, rpL12* (EHI_191750), *rpS14* (EHI_074090), *Cdc2* (L03810), and intron 2 of *ClcB* (EHI_186860), respectively, were designed as described (Vogel et al., 1997). The primers were used in circular RT-PCR using the aforementioned conditions. In some cases, PCR reactions were carried out with the 5′-end radiolabeled Rab2BSs oligonucleotide. Radiolabeling was carried out with the T4 PNK enzyme (New England BioLabs) as suggested by the manufacturer. Where indicated, RT reactions were carried out in the presence of 5 μM Actinomycin D (Sigma-Aldrich), adding the drug immediately after the denaturing step (Houseley and Tollervey, 2010). PCR products were resolved in 8% urea-polyacrylamide gels. The Kappa Syber Fast Universal One-Step qRT-PCR kit (Sigma Aldrich) was used for quantitative RT-PCR with 10 ng of cDNA input in 10 μL.

### SF1, U2AF, and DBR1 plasmid constructs and entamoeba transformants

Using *E. histolytica* gDNA as a template and the respective primer pairs in which the SmaI and XhoI restriction sites were included, the full-length and C-terminus deleted (ΔC) U2AF and Dbr1ΔC, were amplified by PCR and ligated into the SmaI/XhoI-digested pEhExHA expression plasmid able to express N-terminal hemagglutinin (HA)-tagged fusion protein (Saito-Nakano et al., 2007). The wild type and mutant *RabX13* minigenes were purchased to GeneScript (Table S2) and subcloned into pEhExHA. The full-length *EhSF1* gene was amplified by PCR and ligated in inverted orientation into the SmaI/XhoI-digested silencing pKT3M plasmid (Morf et al., 2013). Plasmids were transfected into *E. histolytica* HM1:IMSS trophozoites as described (Nozaki et al., 1999). To avoid lethality, 24 h after transfection the expression of HA-Dbr1ΔC fusion proteins in transient transfectants was induced by the addition of 1.5 μg/mL during 48 h. Stable HA-U2AF, HA-U2AFΔC, and pK-SF1 transfectants were established by culturing in 3, 6, or 10 μg/mL G418 as above. *In vivo* splicing assays were carried out with purified RNA from log phase cultures of transformants to detect *RabX13* unspliced, spliced, lariats and circular RNA variants.

### Western blots

HA-Dbr1, HA-U2AFΔC proteins as well as histone H3 were detected by western blot assays using the anti-HA (Covance) and anti-histone H3 (Cell Signaling) antibodies diluted 1:25,000 and 1:1,000, respectively. SF1 was detected with anti-human SF1 antibodies (Santa Cruz) diluted 1:1,000.

### Statistical analyses

The intensity of each splice isoform was measured using NIH ImageJ software from at least three RT-PCR experiments and analyzed using Student's *t*-test and One-Way ANOVA with SigmaPlot software. For reference, the mean value of each isoform in the absence of Boric acid or at the zero time points was set to 1. qRT-PCR data was analyzed using the 2^−ΔΔC_T_^ method (Livak and Schmittgen, 2001).

## Results

### Identification and validation of full-length intron circular RNAs

**Figure 1:**
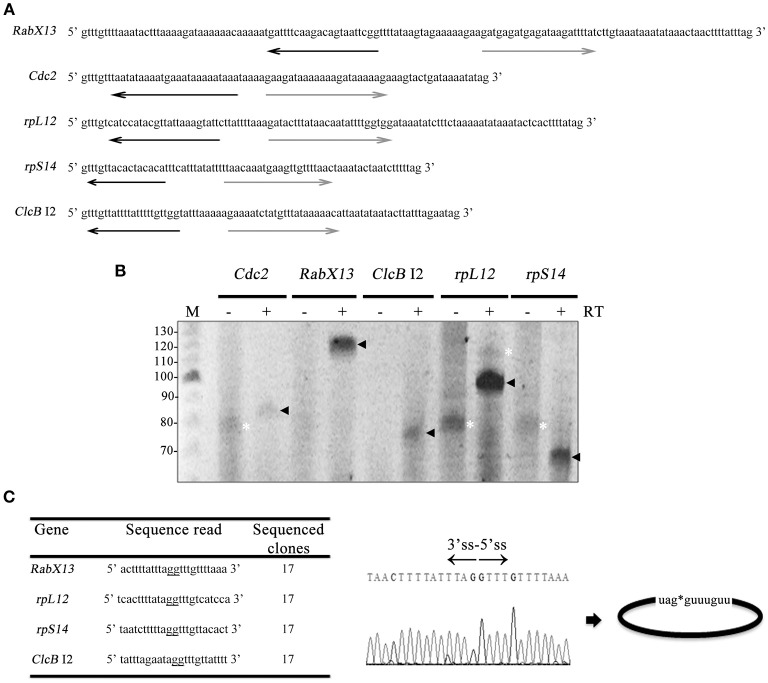
*E. histolytica* full-length intronic circular RNAs. **(A)** Sequences of the introns analyzed in this work. Antisense (black) and sense (gray) outward-facing primers used in circular RT-PCRs. **(B)** 8% urea-polyacrylamide gels showing the amplicons obtained in the circular RT-PCRs alongside a 10 bp ladder (M). Black arrowheads indicate products ~ 5–10 nt larger than the tailed lariats, e.g., flicRNAs. White asterisks indicate products that do not correspond to their respective intron sequences. PCRs with (+) and without (−) RT reactions are indicated. **(C)** Sequence reads of amplicon clones showing ligation points between the 5′ss and the 3′ss (underlined gg residues; also denoted by an asterisk), suggesting full-length intronic circular RNA molecules (right). The electropherogram corresponds to flicRX13.

To detect circular intronic noncoding RNAs, outward facing primers (Figure 1A and Table S1) targeted to five genes of different functions and intron sizes were used in circular RT-PCR (Vogel et al., 1997): the Rab GTPase family member *RabX13* (136 nt intron); the ribosomal protein S14 gene *rpS14* (73 nt intron); the ribosomal protein L12 gene *rpL12* (106 nt intron); the yeast homolog *p34*^*cdc*2^ *Cdc2* (79 nt intron); and the chloride anion channel *ClcB* (57 nt intron 1 and 81 nt intron 2) (Salas-Casas et al., 2006). These genes were selected because their intron size is larger than the mean of *E. histolytica* introns, therefore facilitating their study. Like intron 1 of *ClcB*, the majority of introns initially chosen for this study were too small and/or were A-T rich and were left out because were not amenable to such analyses.

Surprisingly no sequence reads of the introns analyzed corresponded to branched molecules showing a typical miss-incorporation of an A for T at the lariat's BP (Vogel et al., 1997; Barrett et al., 2015). Instead, the sequences corresponded to full-length intronic circularized RNAs (flicRNAs), with ligated G residues of the ends of the introns, 6–15 nt larger than the products expected from the lariat amplicons (Figures 1B,**C**, black arrowheads). No 3′ tail-less lariat structures such as those observed in vertebrates or other types of branched lariats (Li-Pook-Than and Bonen, 2006; Gao et al., 2008; Taggart et al., 2012, 2017; Zhang et al., 2013) were detected. The remaining amplicons (white asterisks) did not match their respective intronic sequences. None of the *Cdc2* reads were informative; therefore, this intron was not analyzed further. Since the *Rabx13* flicRNA (flicRX13) is readily detected in different Entamoeba strains, it was chosen for further characterization and analyses.

**Figure 2:**
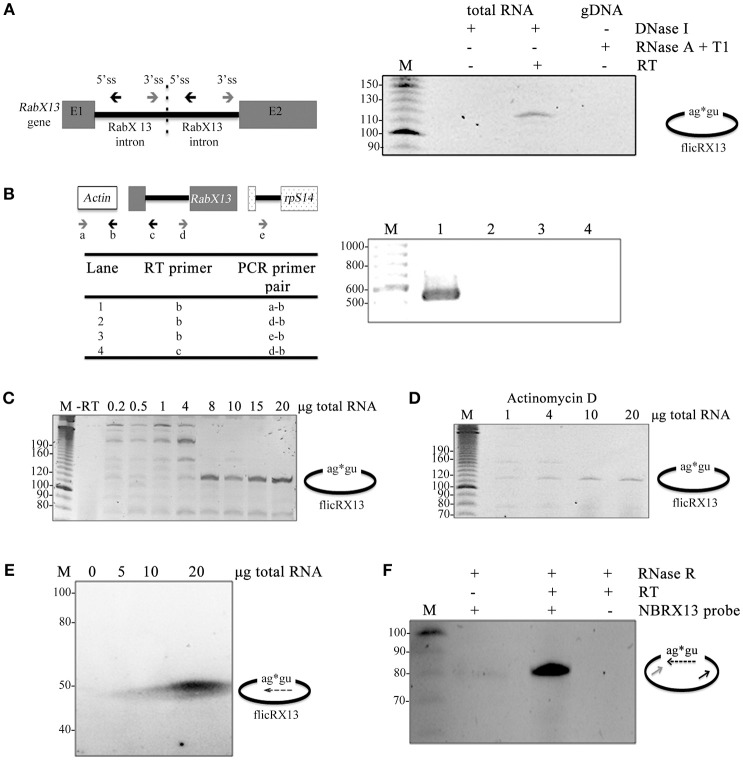
flicRX13 is not a product of intron duplication, trans-splicing, or self-circularization events. **(A)** A diagram of the theoretical intron duplication event is depicted to the left showing the 5′ss, the 3′ss, and the outward facing primers. flicRX13 was detected only in plus RT DNase I-treated total RNA samples suggesting that no tandem templates exist in the genome. E1, exon 1; E2, exon 2. **(B)** Analysis of the possible intermolecular noncanonical trans-splicing events. Details are described in the text. Except for *EhActin* controls (lane 1), no PCR products were detected suggesting that flicRX13 is not a product of either intermolecular trans-splicing nor RT jumps. **(C)** Increasing amounts of total RNA input in the circular RT-PCR experiments proportionally increased flicRX13 amplification, ruling out possible self-ligation events due to dilute RNA samples. **(D)** Addition of Actinomycin D to the RT reaction increased flicRX13 specific amplification as a function of RNA input. **(E)** Different amounts of total trophozoite RNAs were blotted onto nylon membrane and hybridized at full stringency with a radiolabeled probe spanning the splice site ligation points. **(F)** RNase R treated RNA samples were used as input for cDNA synthesis with the antisense primer of Figure 1 in the presence (+) or absence (−) of RT. Circular PCR was carried out with the sense primer and the probe used in **(E)**. Numbers to the left of the gel images indicate the bp ladder (M).

Experiments were carried out to discard any given condition that could render artifactual 5′-3′ss joined amplicons. First, to rule out intron tandem genomic templates, we compared the products obtained in circular RT-PCR experiments using DNase I-treated total RNA, with and without RT, and RNase A+T1-treated gDNA without RT reactions. FlicRX13 was detected only in plus RT DNase I-treated total RNA samples (Figure 2A). Second, to eliminate the possible inter-molecular trans-splicing origin of flicRNAs, cDNA synthesis was carried out with the primer EhActR or Rab2BSas, targeted to the *EhActin* gene and to the *RabX13* intron, respectively. Then, independent PCR reactions were carried out with each cDNA, using the respective reverse primers employed in the RT reaction (EhActR or Rab2BSas) and the forward primers targeted to different transcripts (e.g., *rpS14*) as shown in Figure 2B. Except for the *EhActin* controls (lane 1), no PCR products were detected. Third, to discard possible self-ligation events due to diluted RNA samples, we used increasing amounts of total RNA input in the circular RT-PCR experiments and observed a proportional increase in flicRX13 amplification (Figure 2C). To eliminate artifactual inter- and intra-molecular RT jumps, cDNA was synthesized in the presence of Actinomycin D (Houseley and Tollervey, 2010) with increasing amounts of total RNA inputs. As expected, Actinomycin D increased flicRX13 specific amplification as a function of the RNA input (Figure 2D). Together these experiments suggest that at least flicRX13 does not result from noncanonical trans-splicing events.

To verify the circularity of the intronic RNA molecules, additional criteria were considered. First, stringent northern blot hybridization was performed with a probe spanning the 3′ss G-5′ss G ligation point of flicRX13 (dashed arrow in Figure 2E). Next, total RNA was treated with RNAse R to degrade linear RNA (Suzuki et al., 2006; Burd et al., 2010; Salzman et al., 2012; Jeck et al., 2013; Ashwal-Fluss et al., 2014) and was used to perform circular RT-PCR using primer Rab2BSas (black arrow in Figure 2F) for cDNA synthesis. Only a specific PCR product corresponding to flicRX13 was amplified with the probe and sense primer (dashed and gray arrows respectively). Furthermore, as expected for intronic circular ncRNA (Zhang et al., 2013) we were able to amplify flicRNAs from nuclear RNA and from RNA extracted with miRNA isolation kits. Altogether, our results support the notion that *E. histolytica* flicRNAs are *bona fide* circular intronic RNA molecules.

### Postsplicing origin of flicRNAs

**Figure 3:**
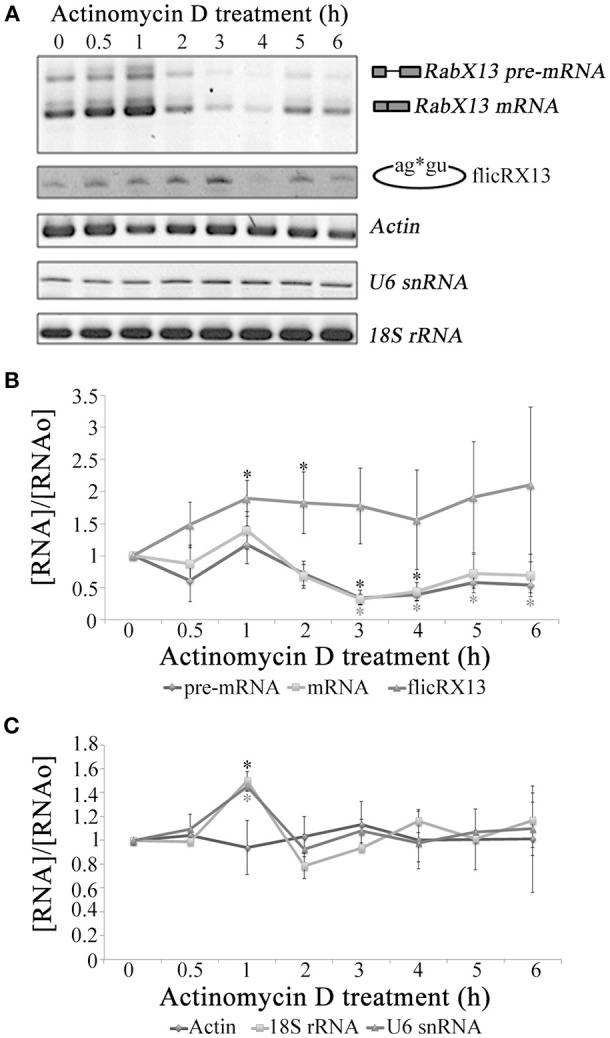
flicRX13 is an RNA processing byproduct and is more stable than its corresponding splicing linear variants. **(A)** *RabX13* gene expression after RNA Pol II inhibition by Actinomycin D. As the loading control *EhActin* gene expression was analyzed. Pol I (18S rRNA) and Pol III (U6 snRNA) inhibition by Actinomycin D was also monitored. **(B)** Quantitative analysis of RabX13 gene expression after RNA Pol II inhibition by Actinomycin D shown in **(A)**. The increase of flicRX13 expression at 1 and 2 h posttreatment is significantly different (t-Student *P* < 0.0061; *P* < 0.0041, respectively) as well as the decrease in pre-mRNA and mRNA (t-Student *P* < 0.001 at 3 h; black and gray asterisks, respectively). **(C)** Quantitative analysis of 18S rRNA, U6 snRNA and *EhActin* gene expression after RNA Pol II inhibition by Actinomycin D shown in **(A)**. The increases in 18S rRNA and U6 snRNA (black and gray asterisks, respectively) are statistically significative (t-Student *P* < 0.001).

Circular RNA molecules are more stable than their corresponding splicing variants (Jeck et al., 2013). This is also true for flicRX13, since this product appears early in the growth phase and is maintained throughout a 72-h standard growth culture (not shown) incubated in the presence of Actinomycin D at different time points. After 3 h of transcription inhibition, *RabX13* pre-mRNA and spliced variants nearly disappear while the flicRNA population remained up to 6 h of treatment (Figures 3A,B). As previously observed (López-Camarillo et al., 2003), the expression of *EhActin* remained unchanged. U6 snRNA and 18S rRNA were also not affected, indicating that RNA polymerases III (Miranda et al., 1996) and I (Oakes et al., 1993), respectively, were not affected (Figure 3C). These results show the different stability between pre-mRNA, mRNA and flicRX13, and that the half-life of flicRX13 is greater than that of its parental mRNA.

**Figure 4:**
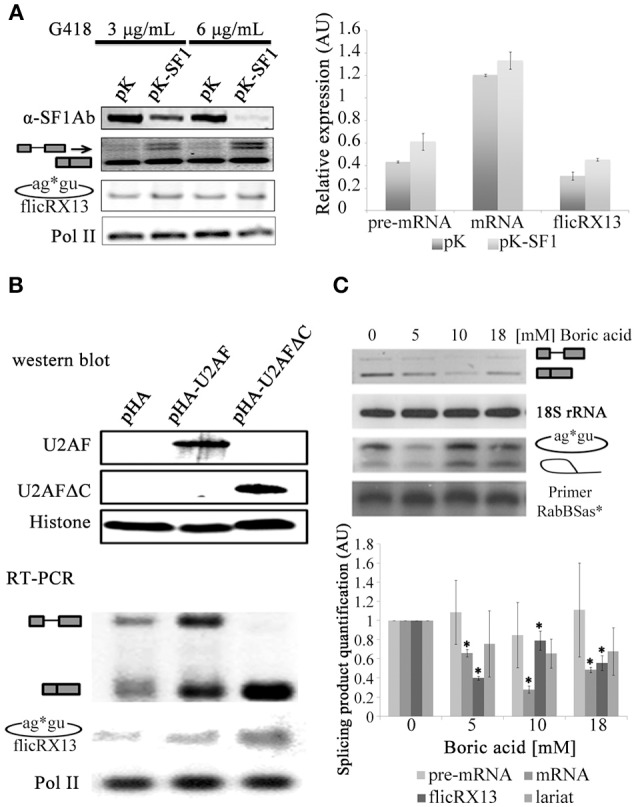
Inhibition of the second step of splicing alters flicRNA production. **(A)** Stable SF1-silencing (pK-SF1) and empty pKT3M vector (pK) transfectants were established at 3 and 6 μg/mL G418. Silencing was assessed by western blot (top) achieving 40–90% efficiency. Absence of SF1 slightly increased pre-mRNA/unspliced and mRNA *Rabx13* transcripts (middle), and flicRX13 production (bottom); however, such increments were not statistically significant (right plot). **(B)** Amoeba transformants overexpressing wt (HA-U2AF) and carboxy-terminal deleted (HA-U2AFΔC) U2 auxiliary splicing factor were established. Appropriate expression of the hemagglutinin-U2AF fusion proteins were monitored and compared to histone H3 expression by western blot (top). Compared to the empty Entamoeba HA expression plasmid (pHA) pHA-U2AF increased the overall expression of all *RabX13* transcription products. However, HA-U2AFΔC substantially increased mRNA (spliced) products and flicRX13. RNA polymerase II expression was equivalent between the transformants. **(C)** Expression of the *RabX13* gene transcript variants (unspliced, spliced, flicRX13 and intron lariat) after incubation of *E. histolytica* trophozoites with different concentrations of boric acid, an inhibitor of the second step of splicing. 18S rRNA expression was used for normalization purposes. To detect intron lariats, circular RT-PCR was carried out with the ^32^P-labeled Rab2BSas primer (Rab2BSas*), which was used as the loading control. Bottom panel, products in the upper panel were quantitated and statistically analyzed. Decrease of spliced *RabX13* variant and flicRX13 were statistically significant (ANOVA *P* ≤ 0.001, asterisks in bold). Values are the average of three independent experiments.

To explore the splicing or postsplicing origin of flicRNAs we first silenced the E complex factor SF1 (Loftus et al., 2005; Valdés et al., 2014) and analyzed flicRX13 abundance in the transfected amoebas. After confirming SF1 mRNA depletion by RT-PCR, we observed that reduced levels of SF1 moderately increased intron retained/spliced precursor and RabX13 spliced variants but not flicRX13, demonstrating its role in splicing, but not in flicRNA production (Figure 4A). We next overexpressed the E complex U2 auxiliary splicing factor, U2AF. Full-length U2AF slightly increased flicRNA formation in response to increased overall synthesis of unspliced and spliced RabX13 transcripts; however, overexpression of the C-terminus deleted U2AF strongly increased both splicing and flicRNA production (Figure 4B), suggesting that flicRNAs are byproducts of splicing after complex E has been assembled.

To seek whether flicRNA formation occurs during or after the first step of splicing, we compared the abundance of unspliced pre-mRNA/intron retained, spliced mRNA, lariats and flicRNAs in RNA extracts from Entamoeba cultured in boric acid, an inhibitor of the second step of splicing (Shomron and Ast, 2003). By circular RT-PCR using a radioactive primer, we detected *RabX13* and *rpS14* lariats with the predicted BP (Figure S1 and Figure 4C) and with the expected 6–11 nt size, which is smaller compared to flicRNAs. As expected, 5 mM boric acid significantly affected the amplification of all splicing products (ANOVA *P* ≤ 0.001), particularly flicRX13. However, with 10 mM boric acid, a slight recovery of flicRX13 and lariat molecules (including actual and cryptic BP lariats, and lariat-3′ exon intermediaries) was observed at the expense of the spliced products (Figure 4C). These results suggest that flicRX13 is another final product of splicing, most likely originated by the circularization of lariats. Because *in vivo* boric acid treatment is a novel experimental approach, the accumulation of lariats in boric acid treated mammalian cell cultures was validated as well. Lariats of the HPV16 proximal 3′ss accumulated in infected HeLa cells only (Figure S1).

### Elements and factors involved in flicRNAs biogenesis

Structurally, flicRNAs resemble circular group I and group II introns. We did not explore the possible origin of flicRNAs by self-splicing reactions homologous to group I introns because *E. histolytica* introns have no discernible internal guide sequences that facilitate self-splicing (Winter et al., 1990), and we did not observe circle ligation points containing guanine triplets, that would result by the attack of an exogenous αG to the 5′ss, followed by the 5′ exon attack to the 3′ss as previously reported (Hausner et al., 2014). To explore whether flicRNAs are self-spliced such as group II introns, synthetic radio-labeled RabX13 and ai5γ group II intron transcripts were incubated in the appropriate buffers. Whereas the intron-3′ exon intermediate and mRNA were produced from the ai5γ intron transcript, only intact RabX13 transcripts were observed suggesting that flicRNAs might not originate through this mechanism (Figure S2). However, since RabX13 intron does not have the RNA elements required for auto excision (data not shown), we cannot completely discard this route of intron circularization in *E. histolytica*.

**Figure 5:**
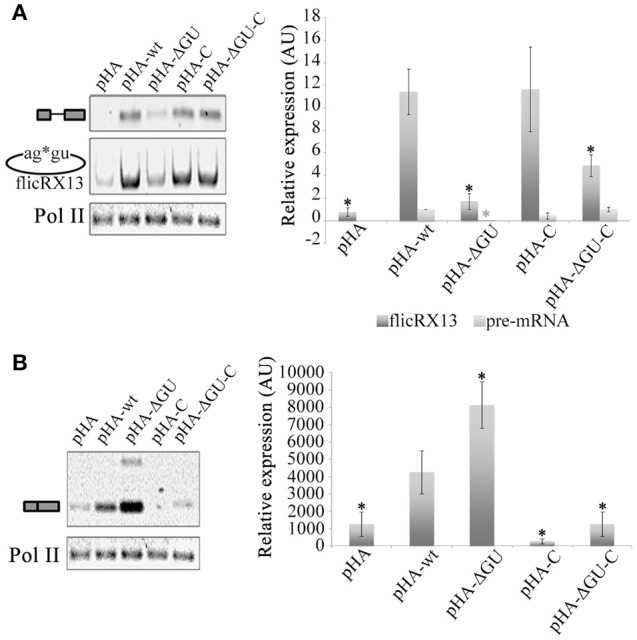
The GU-rich 5′ss element of the intron is involved in flicRNA biogenesis and function. Amoeba transformants expressing wt, 5′ss mutant (GUUUGUUU to GUAAGAA), C-enriched BP mutant (AACTAACTTTT**A** to ACCTCCCTCTT**A**; A in bold corresponds to the BP mapped in Figure S1), and double mutant *RabX13* minigene constructs were established, and the impact on unspliced, spliced, and flicRX13 *RabX13* variant expression was assessed by qRT-PCR using primers CS+116 and Rab-REx2 targeted to plasmid sequences **(A)** and endogenous *RabX13* mRNA **(B)**. Decreases of plasmid-derived unspliced *RabX13* variants and flicRX13 were statistically significant; likewise, the increase of endogenous mRNA was statistically significant (ANOVA black asterisk *P* ≤ 0.0001, gray asterisk *P* ≤ 0.01, respectively). Values are the average of three independent experiments.

To escape from the debranching reaction, ciRNA biogenesis requires a GU-rich 5′ss and a C-rich element upstream of the BP (Zhang et al., 2013). Therefore, to explore whether these elements influence flicRNA formation, minigene intron Entamoeba transfectants were established harboring the wt intron, a minus GU-rich 5′ss (ΔGU) intron, an intron containing a C-rich element 11 nt upstream of the BP (C; uncommon in the Entamoeba genome), and an intron with both modifications (ΔGU-C), all of which are flanked with partial exon sequences tagged with plasmid-specific sequences. The different RNA species transcribed from the transfected plasmids were monitored by qRT-PCR with primers CS+116 and Rab-REx2. The elimination of the GU-rich element at the 5′ss affected flicRNA formation even in the presence of the synthetic C-rich element. The C-rich element alone has a similar effect but not as strong as the 5′ss-GU element indicating that the GU-rich element is sufficient to promote intron circularization (Figure 5A).

**Figure 6:**
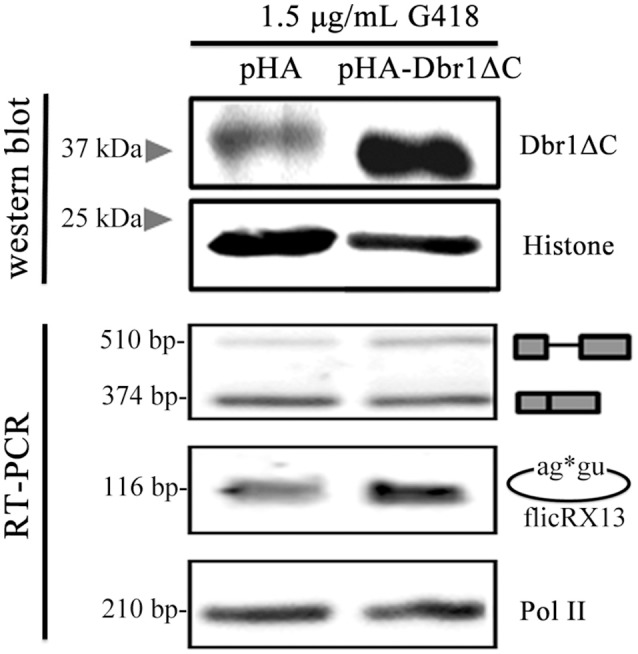
flicRX13 is a postsplicing product: Dbr1 is involved in Entamoeba flicRNA production. *E. histolytica* trophozoites were transiently transfected with the empty vector pEhExHA (pHA), or with the C-terminus deleted Dbr1 (pHA-Dbr1ΔC) plasmids and 24 h latter were tested under low selection pressure (1.5 μg/mL G418). Overexpression of Dbr1 (wt or ΔC) was monitored by western blot using anti-HA antibody compared to anti-histone H3 (loading control). The effect of Dbr1 (wt or ΔC) overexpression on flicRX13, *RabX13* mRNA, and RNA Pol II (loading control) gene expression was analyzed by RT-PCRs.

Dbr1 participates in sisRNAs and ciRNAs biogenesis (Zhang et al., 2013; Talhouarne and Gall, 2014). To explore the possible *E. histolytica* Dbr1 involvement in flicRNAs biogenesis, we attempted siRNA-mediated Dbr1 silencing without success. Also, the establishment of the catalytically deficient Dbr1 (Dbr1ΔC) overexpressor amoebas was unsuccessful. Therefore, *in vivo* splicing assays were conducted using Dbr1ΔC transiently transfected trophozoites under low selection pressure (1.5 μg/mL G418). After assessing Dbr1ΔC expression by western blot, the different *RabX13* splicing products were monitored (Figure 6). Surprisingly, compared to the control Dbr1ΔC overexpression increased flicRNA levels. Taken together, our data indicate that *E. histolytica* flicRNAs originate from lariat molecules after the second step of splicing has taken place, and that the GU-rich 5′ss and Dbr1 are implicated in their biogenesis. However, the implication of Dbr1 in this process might be indirect or require additional factors.

### Insights into flicRNAs possible functions

circRNAs bound to the U1 snRNA promote the transcription of their parental genes, which interact with the RNA polymerase II at the promoter or during elongation (Zhang et al., 2013; Li et al., 2015). Our experimental design with the minigene constructs allowed us to explore the possible function of Entamoeba flicRNAs irrespective of the snRNA involved. Again, *in vivo* splicing assays were performed using RNA from minigenes transformed amoebas and the different endogenous RNA species were monitored. Unexpectedly, mutations in the 5′ss-GU element resulted in an increase of endogenous *RabX13* expression (Figure 5B), indicating that such elements of flicRX13 silences the expression of its parental gene in *cis*.

**Figure 7:**
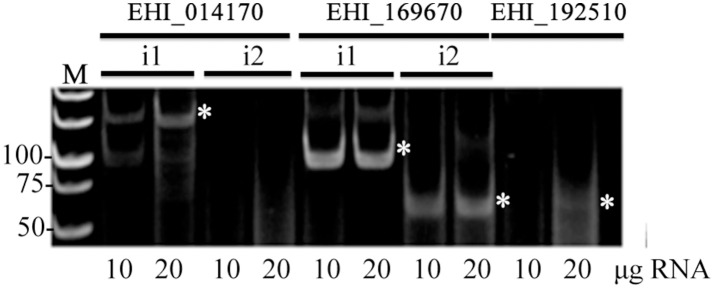
Introns of some virulence-related genes also render flicRNAs. Appropriate sets of outward-facing primers were designed to amplify virulence-related flicRNAs (white asterisks) by circular RT-PCR as in Figure 2F. Different amounts of small RNA input isolated with the miRNA isolation kit were used. Gene accession numbers are indicated; i1, intron 1; i2, intron 2. Numbers to the left indicate the bp ladder (M).

Finally, to explore the general nature of flicRNAs in the Entamoeba ncRNA repertoire, we sought additional flicRNAs, particularly those involved in liver abscess formation (Meyer et al., 2016). We detected flicRNAs from the gene products in the loci EHI_014170 (intron 1), EHI_169670 (both introns), and the mono-intronic EHI_192510 (Figure 7), suggesting that flicRNAs are common ncRNA species in *E. histolytica* involved in gene expression regulation.

## Discussion

Here, we identified full-length intronic circular RNAs or flicRNAs, whose 5′ss is ligated to the 3′ss, in the protozoan parasite *E. histolytica*, which differs from other parasitic or mammalian circular RNAs. For example, *D. discoideum* and *P. falciparum* circular RNAs contain exons and some, are related to exon-skipping splicing events (Wang et al., 2014), indicating that in protozoa there might exist a wide repertoire of circular noncoding RNAs that originated by distinct mechanisms in protozoa.

### flicRNAs biogenesis in *E. histolytica*

Exon-intron-containing circular RNAs are generated by backsplicing, mainly favored by protein-mediated or sequence-mediated approximation of intronic repetitive sequences that flank nonconsecutive circularized exons (Ashwal-Fluss et al., 2014; Jeck and Sharpless, 2014; Conn et al., 2015; Ivanov et al., 2015). The molecular mechanisms involved are not fully understood, but it is clear that circRNA formation competes with splicing (Ashwal-Fluss et al., 2014) and, in spite of the lack of direct biochemical evidence (Barrett et al., 2015), the occurrence of some circular RNAs have been correlated to exon-skipping splicing events (Zaphiropoulos, 1996; Surono et al., 1999; Zhang et al., 2014), as reported for the exon-derived circular RNAs found in *D. discoideum* and *P. falciparum* (Wang et al., 2014). The biogenesis of *E. histolytica* flicRNAs characterized here does not depend on complex E splicing formation, possibly due to the strong (Larson and Hoskins, 2017) and most frequent Entamoeba 5′ss (GUUUGUU) (Davis et al., 2007; Hon et al., 2013) since SF1 is not related to splice site definition nor to flicRNA formation. The impact of the U2 auxiliary factor in flicRNA formation indicates a closer relationship between complex A factors and the requirements for intron circularization, particularly those recruited with the entrance of the U2 snRNP, facilitated by U2AF, that not only defines the 3′ss (Shepard et al., 2002; Förch and Valcarcel, 2003), but also contacts both RNA polymerase II and the nineteen complex (David et al., 2011), which is part of the spliceosomal intron lariat complex, along with other components of the U2 snRNP, and remain attached to the lariat after spliceosomal disassembly (Yoshimoto et al., 2009; Fourmann et al., 2013). Likewise, such impact of U2AF on flicRX13 formation supports the notion that flicRNAs originate by postsplicing events, ruling out the autosplicing route of flicRNA biogenesis.

The circular RNAs in *E. histolytica* are similar to some human ncRNAs. They are full-length intronic circles with joined 5′ and 3′ss and are, thus flicRNAs. Although functionally related, mammalian EIcircRNAs are dissimilar to flicRNAs since they contain both intronic and exonic sequences (Li et al., 2015). Circular ncRNAs are structurally similar to flicRNAs, which are 1) the tail-less ciRNAs consisting of circular intron lariats derived from splicing, which escape lariat-debranching enzyme activity, due to a 7 nt GU-rich element at the 5′ss and a C-rich element 11 nt upstream of the BP (Zhang et al., 2013), and 2) the senescence-related and translation-inhibiting human intron-originated circular RNAs, similar to cirRNAs and flicRNAs, whose biogenesis remains unknown (Taggart et al., 2012, 2017; Abdelmohsen et al., 2017; Panda et al., 2017).

C-rich elements are absent in the genome and the BP environment of Entamoeba introns explaining why even the addition of synthetic C elements fails to elicit flicRNA production. The closest resemblances of such elements are the NYYU*A*Y pyrimidine-rich elements in the vicinity of the BP (Wilihoeft et al., 2001), suggesting that other cis elements are necessary for their biogenesis. The GUUUGUU 5′ss consensus sequence of *E. histolytica* introns resembles the GU-rich element of ciRNAs. Here, we showed that nucleotide substitutions of this 5′ss for a vertebrate 5′ss consensus (GUAAGAA) almost abolishes the formation of flicRNAs, suggesting that such GU-rich elements are essential for splicing and flicRNA biogenesis, probably by allowing escape from the debranching reaction. Interestingly, the artificial introduction of a C-rich BP element had no ostensible effect in flicRNA formation indicating that the Entamoeba genome adapted to a C-poor environment to carry out flicRNA biogenesis using only strong 5′ss GU-rich elements. During splicing complex E formation, U1 snRNA binds to the 5′ss (Larson and Hoskins, 2017). In *E. histolytica*, the U1 snRNP proteins U1A, U1C, and U170k are able to recruit the pre-mRNA processing machinery (Valdés et al., 2014) even in the absence of the U1 snRNA (Dávila López et al., 2008). However, as reported in yeast and *in vitro* systems (Kandels-Lewis and Seraphin, 1993; Rhode et al., 2006), it is possible that the Entamoeba U6 snRNA activates the 5′ss and splicing due to their high degree of base complementarity. Therefore, compared to the wild type 5′ss the changes introduced in the ΔGU mutant elicited a different splicing response due to impaired 5′ss activation. The link of the GU-rich element to debranching reaction escape is still under investigation.

Taggart and coworkers detected the first intron-derived circular molecules similar to flicRNAs by deep sequencing (Taggart et al., 2012, 2017). They observed that approximately 3% of the BP corresponded to the 3′ss of the respective transcripts, thus circular 5′-3′ss ligated molecules, and, although they did not characterize such molecules further, they proposed their postsplicing origin either by a third nucleophilic attack or by a debranching-ligation event. Our results using boric acid as the second step of splicing inhibitor (Shomron and Ast, 2003) suggest a postsplicing origin of flicRX13. We cannot discard that flicRNAs may originate via a third nucleophilic attack as proposed for Group II introns (Murray et al., 2001). However, a more complex debranching-ligation reactions scenario is more likely to occur since it must involve additional factors. Whereas in situations where 2′-5′ phosphodiester bond hydrolysis is impaired, such as in human and yeast Dbr1-deficient cells, intron lariats accumulate (Montemayor et al., 2014; Han et al., 2017), overexpression of an equally impaired Dbr1 in amoeba transformants, unexpectedly increased flicRX13 formation, strengthening the notion that flicRNAs originate from accumulated lariats and that other factors might participate in circularization of Entamoeba introns.

BP conformation is essential for stable recognition and catalysis by Dbr1 CTD and lariat recognition loop (LRL) domains; therefore it is possible that CTD-less Dbr1 might have hydrolyzed the BP and, due to the unstable LRL-BP interaction and the 5′ss GU-rich element, the intron was now exposed to yet to be identified circularization factors, RNA ligase for example, in addition to the intron lariat large complex (U2, U5 and U6 snRNPs, Ntr1, Prp43, and Dnr1) (Yoshimoto et al., 2009; Fourmann et al., 2013; Garrey et al., 2014). Finally, removal of the 3′ tail of the lariat precedes debranching (Chapman and Boeke, 1991; Salem et al., 2003), and we detected that the 3′ss of RabX13 and rps14 lariats are less than 10 nt downstream of the BP, placing them within the LRL-BP recognition groove and protecting them from exonucleolytic degradation. Altogether, these observations support the role of Dbr1 in flicRNA biogenesis and suggest that debranching limits access/competes with other necessary factors for intron circularization. We cannot rule out the possibility that flicRNAs arise from a third nucleophilic attack even though such reaction has not been reported in postsplicing events.

### flicRNAs possible functions

Cytoplasmic exon-containing circRNAs function as miRNA sponges (Jeck and Sharpless, 2014; Chen, 2016). Conversely, the small (thus without space to accommodate miRNA target sites), and nuclear *E. histolytica* flicRNAs most likely have different functions. It is currently accepted that intron containing linear and circular RNAs are retained in the nuclei by a similar mechanism (Chen, 2016). Although by mechanisms not fully understood, intronic circular RNAs participate in gene transcription regulation (Chen, 2016). CLIP-Seq experiments identified intronic circular RNAs associated with Pol II (Li et al., 2015), and the depletion of the abundantly expressed ciRNAs such as ci-ankrd52 and ci-sirt7 results in lower transcription of their parental genes (ANKRD52 y SIRT7) indicating that they promote their transcription by interacting with the Pol II elongation complex (Zhang et al., 2013). Other nuclear circRNAs interact with the U1 snRNP and Pol II on the promoter of their parental genes, stimulating their transcription. Both knockdown of these circRNAs as well as disruption of U1 snRNA-circRNA binding with morpholinos anti-U1 diminishes transcription of their parental genes (Li et al., 2015). In contrast, expression of ΔGU minigenes showed that Entamoeba flicRX13 apparently inhibits transcription of its parental gene, in which the 5′ss GU-rich element again seems to be actively involved. Although direct evidence of flicRNA-RNA Pol II interaction still lacks, our findings suggest an inhibitory role of flicRNAs on transcription. Recently, it has been shown that small regulatory RNAs inhibit RNA polymerase during elongation in the nematode *C. elegans* (Guang et al., 2010). Interestingly the addition of a C-rich BP element positively affected transcription in *cis* probably due to poly C binding hnRNPs (Chaudhury et al., 2010; Han et al., 2010), which are able to bind to such elements in the transcripts, favoring their expression and transcription over other genes (Kim et al., 2005; Meng et al., 2007).

Current experiments are directed to support our present models in which silencing of *Rabx13* transcription might occur via flicRX13-U6 snRNA binding or flicRNA-U1A/TIA-1/TIAR binding prior interaction with Pol II at the promoter or during elongation. In conclusion flicRNAs are common ncRNA species in *E. histolytica* that are involved in gene expression regulation of different regulatory processes including virulence traits.

## Author contributions

MM-F, EA-M, NV-S, and JV conception and experimental design. MM-F, EA-M, CV, EA-L, SZ, NV-S, OS-C, and JV data acquisition and interpretation. MM-F and JV manuscript preparation.

### Conflict of interest statement

The authors declare that the research was conducted in the absence of any commercial or financial relationships that could be construed as a potential conflict of interest. The reviewer MDCG declared a shared affiliation, with no collaboration, with several of the authors, MM-F, EA-M, CV, JV, NV-S, to the handling Editor.
