# Supplementary material for: Postsplicing-Derived Full-Length Intron Circles in the Protozoan Parasite Entamoeba histolytica
Source: Front Cell Infect Microbiol. 2018 Aug 3;8:255. doi: 10.3389/fcimb.2018.00255 (PMC6085484; doi:10.3389/fcimb.2018.00255)
Supplement: Supplementary file 1 [file Table_1.DOCX]

**Table S1:** Genes, oligonucleotides and conditions used in the experiments described in the main text. Gene accession numbers are in parenthesis. All amplifications were done at 35 cycles, except that for Dbr1 which was carried out at 40. US, unspliced; S, spliced.

| **Gene** | **Oligonucleotide** | | **PCR conditions** | **Amplicon size** |
| --- | --- | --- | --- | --- |
|  | **Name** | **Sequence (5’ 🡺 3’)** |  |  |
| *RabX13*  (EHI_065790) | Rab2 F | cgttgttggagactcttcagttgg | 94°C/45’’, 60°C/45’’, 72°C/1’ | US = 510 bp  S = 374 bp |
|  | Rab2 R | gacccatttcagttgaaacagttc |  |  |
|  | Rab2BSs | gatgagatgagataagattttatc | 94°,45’’; 53°,45’’; 72°C,30’’ | Lariat ≈ 105 bp  flicRX13 = 116 bp |
|  | Rab2BSas | ccgaattactgtcttgaaaatc |  |  |
|  | R2T7F | taatacgactcactatagagatatgaagtagatggaacg | 94°C,45’’; 55°C,45’’; 72°C,1’ | 401 bp  (When used with Rab2 R) |
|  | Rab2Rex 2 | ttgaacaccacgataataactagt |  |  |
|  | NBRX13 | cttttaaagtatttaaaacaaacctaaataaaagttagtttatatttatt | 94°C,45’’; 55°C,45’’; 72°C,30’’ | 79 bp  (When used with Rab2BSs) |
| *rpL12*  (EHI_191750) | L12BSs | gatactttataacaatattttggtgg | 94°C,45’’; 56°C,45’’; 72°C,30’’ | Lariat ≈ 87 bp |
|  | L12BSas | gaatactttaataacgtatggatg |  |  |
| *rpS14*  (EHI_074090) | S14BSs | ttaacaaatgaagttgttttaac | 94°C,45’’; 51°C,45’’; 72°C,30’’ | Lariat ≈ 57 bp  flicS14 = 67 bp |
|  | S14BSas | aatgaaatgtgtagtgtaacaaac |  |  |
| *Cdc2*  (L03810) | Cdc2BSs | gaagataaaaaaagataaaaag | 94°C,45’’; 48°C,45’’; 72°C,30’’ | Lariat ≈ 65 bp |
|  | Cdc2BSas | atttatttttatttcattttatatt |  |  |
| *ClcB*  (EHI_186860) | CloBSs | gaaaatctatgtttataaaaac | 94°C,45’’; 47°C,45’’; 72°C,30’’ | Lariat ≈ 50 bp |
|  | CloBSas | ccaacaaaaataaaataacaaac |  |  |
| *U6 snRNA*  (EHU43841) | EhU6 F | ggatccacttcggtggaaat | 94°C/45’’, 62°C/45’’, 72°C/30” | 92 bp |
|  | EhU6 R | cttctcgtatgagcgtgtcatc |  |  |
| *DBR1*  (EHI_062730) | EhDBR S | atggcaactgaacaaatacaac | 95°C/45’’, 58°C/45’’, 72°C/1’ | 882 bp |
|  | EhDBR AS | gtcagggaattgtgctttatttg |  |  |
|  | XhoI-asDbrF | gtaactcgagtcaatggttgtcaaacaaatgtaa |  | 1108 bp (when used with EhDBR S) |
| *18S rRNA*  (AB426549) | FRibEh239F | attgtcgtggcatcctaactca | 94°C/1’, 62°C/1’, 72°C/1’ | 173 bp |
|  | RRibEh88R | gcggacggctcattataaca |  |  |
| *EhActin*  (EHI_107290) | EhActF | gagctgtattcccatccattgttg | 94°C/45’’, 65°C/45’’, 72°C,45’’ | 535 bp |
|  | EhActR | ctttcagcagtagtggtgaaagc |  |  |
| *EhRNA Polymerase II*  (EHI7A_158420) | RNAPIIs | gatccaacatatcctaaaacaaca | 94°C/45’’, 60 °C/45’’, 72 °C/45’’ | 210 bp |
|  | RNAPIIas | tcaattattttctgacccgtcttc |  |  |
| *EhSF1*  (EHI_193510) | EhSF1F | cacaccccgggatggaagaagtaaaagaagttgattataaa | 94°C/30’’, 55 °C/30’’, 72 °C/30’’ | 930 bp |
|  | EhSF1R-Xho | gtaactccgagtcacataaactcatcaaatgccctc |  |  |
