# Supplementary material for: Postsplicing-Derived Full-Length Intron Circles in the Protozoan Parasite Entamoeba histolytica
Source: Front Cell Infect Microbiol. 2018 Aug 3;8:255. doi: 10.3389/fcimb.2018.00255 (PMC6085484; doi:10.3389/fcimb.2018.00255)
Supplement: Supplementary file 2 [file Table_2.DOCX]

**Table S2:** Minigene sequences. Exons and introns are indicated in capital and lower case letters, respectively. Mutations are shown in square brackets. RabRex 2 oligonucleotide complementary sequence is shown in capital bold letters. HindIII cut site sequence is shown in cursives. XmaI (cccggg) and XhoI (ctcgag) sites are underlined.

| **Sequence name** | **Sequence** |
| --- | --- |
| **Rab WT-HindIII** | **cccggg**AAAAGATATGAAGTAGATGGAACGACATATAAAGTTCAAATATGGGATACTGgtttgttttaaatactttaaaagataaaaaacaaaaatgattttcaagacagtaattcggttttataagtagaaaaagaagatgagatgagataagattttatct*ttcgaa*aataaatataaactaacttttatttagCTGGACAAGAAAAATTTAGAACTATTATT**ACTAGTTATTATCGTGGTGTTCAAtgactcgag** |
|  |  |
| **Rab Box ∆GU-HindIII** | **cccggg**AAAAGATATGAAGTAGATGGAACGACATATAAAGTTCAAATATGGGATACTGgt[aa]g[aa]ttaaatactttaaaagataaaaaacaaaaatgattttcaagacagtaattcggttttataagtagaaaaagaagatgagatgagataagattttatct*ttcgtaa*aataaatataaactaacttttatttagCTGGACAAGAAAAATTTAGAACTATTATT**ACTAGTTATTATCGTGGTGTTCAATGActcgag** |
|  |  |
| **Rab Box C-HindIII** | **cccggg**AAAAGATATGAAGTAGATGGAACGACATATAAAGTTCAAATATGGGATACTGgtttgttttaaatactttaaaagataaaaaacaaaaatgattttcaagacagtaattcggttttataagtagaaaaagaagatgagatgagataagattttatct*ttcgaa*aataaatataa[c]ct[cc]ct[c]ttatttagCTGGACAAGAAAAATTTAGAACTATTATT**ACTAGTTATTATCGTGGTGTTCAAtgActcgag** |
|  |  |
| **Rab Box ∆GU/Box C-HindIII** | **cccggg**AAAAGATATGAAGTAGATGGAACGACATATAAAGTTCAAATATGGGATACTGgt[aa]g[aa]ttaaatactttaaaagataaaaaacaaaaatgattttcaagacagtaattcggttttataagtagaaaaagaagatgagatgagataagattttatct*ttcgtaa*aataaatataa[c]ct[cc]ct[c]ttatttagCTGGACAAGAAAAATTTAGAACTATTATT**ACTAGTTATTATCGTGGTGTTCAAtgactcgag** |
|  |  |
