# Supplementary material for: Postsplicing-Derived Full-Length Intron Circles in the Protozoan Parasite Entamoeba histolytica
Source: Front Cell Infect Microbiol. 2018 Aug 3;8:255. doi: 10.3389/fcimb.2018.00255 (PMC6085484; doi:10.3389/fcimb.2018.00255)
Supplement: Supplementary file 3 [file Image_1.pdf]

Figure S1

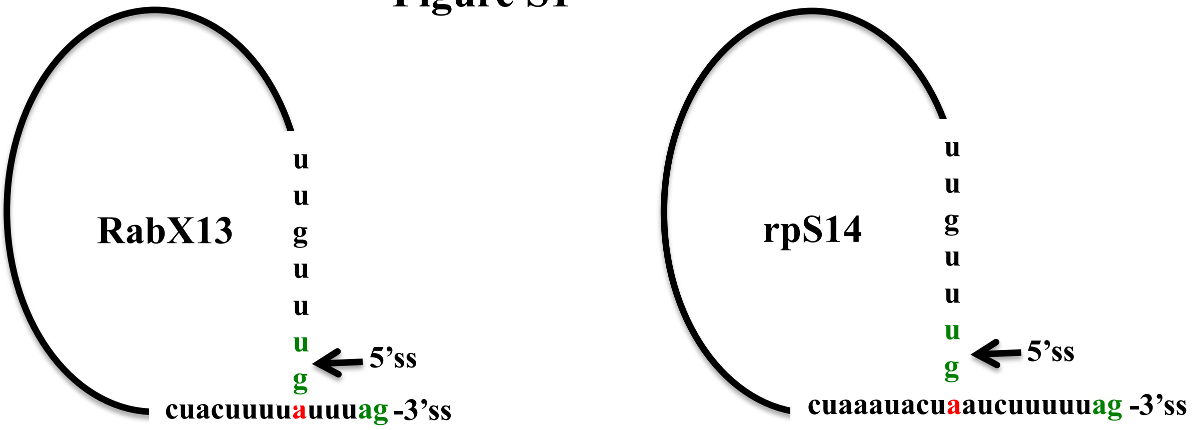

RabX13 and rpS14 lariat structures. Structures were inferred from sequencing of RT-PCR amplicons obtained from RNA of *E. histolytica* trophozoites incubated with 5 mM boric acid. The 5'ss (5'**gu**uuguu), the 3'ss (**ag**), and the branch point (BP) are indicated. BPs are localized 6 and 11 nt upstream the 3'ss of RabX13 and rpS14, respectively. During clone sequencing, a "t" was read in the position of the red "a" as expected for the presence of a ramification (Vogel et al., 1997).

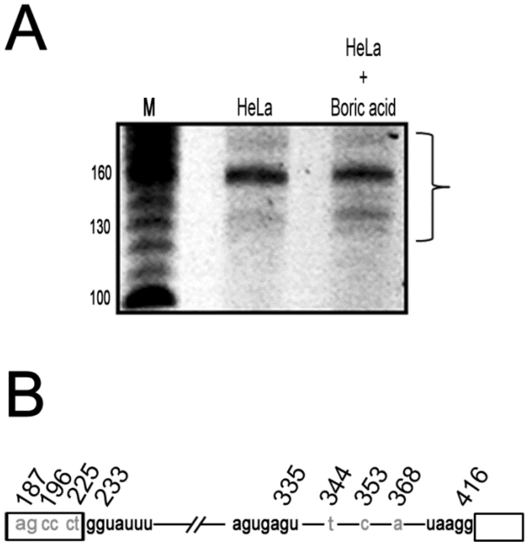

Impact of boric acid treatment on HPV-18 E6 branch point selection. A) Representative gel showing amplicons obtained in the HPV-18 E6 cRT-PCR alongside a 10 bp ladder (M). Bands comprised in the bracketed region were cloned and sequenced. **B)** Diagram of the E6 intron with its flanking exons. Black letters indicate the 5'ss, the canonical BP[7], and the 3'ss. Boric acid treatment of HeLa cells enriched alternative branch points (in gray) localized between the canonical BP and the 3'ss at positions 344, 356 and 368 as of GenBank numeration (Accession number NC_001357). Also, alternative 5'ss were detected at positions 187, 196 and 225.

Methods for Figure S1

Human cervical carcinoma (HeLa) cells infected with the Human Papilloma Virus 18 (HPV-18) were grown in DMEM (Life Technologies) supplemented with 10 % fetal bovine serum and incubated at 37 °C in a humid atmosphere of 5% CO_2. At 80% confluence, cultures were incubated (or not) with 5 mM boric acid during 1.5 hours[8] and after that, total RNA was isolated by the TRIzol method. Outward facing primers matching the HPV-18 E6 intron (Table S1) were designed and used in a cRT-PCR in order to detect the branch point of the E6 intron. Then amplicons were isolated, cloned and sequenced.

References

[7]De la Rosa-Ríos MA, Martínez-Salazár M, Martínez-García M, González-Bonilla C, Villegas-Sepúlveda N. 2006. The intron 1 of HPV 16 has a suboptimal branch point at a guanosine. *Virus Res* 118: 46-54.

[8]Shomron N, Ast G. 2003. Boric acid reversibly inhibits the second step of pre-mRNA splicing. FEBS letters 552:219-224.

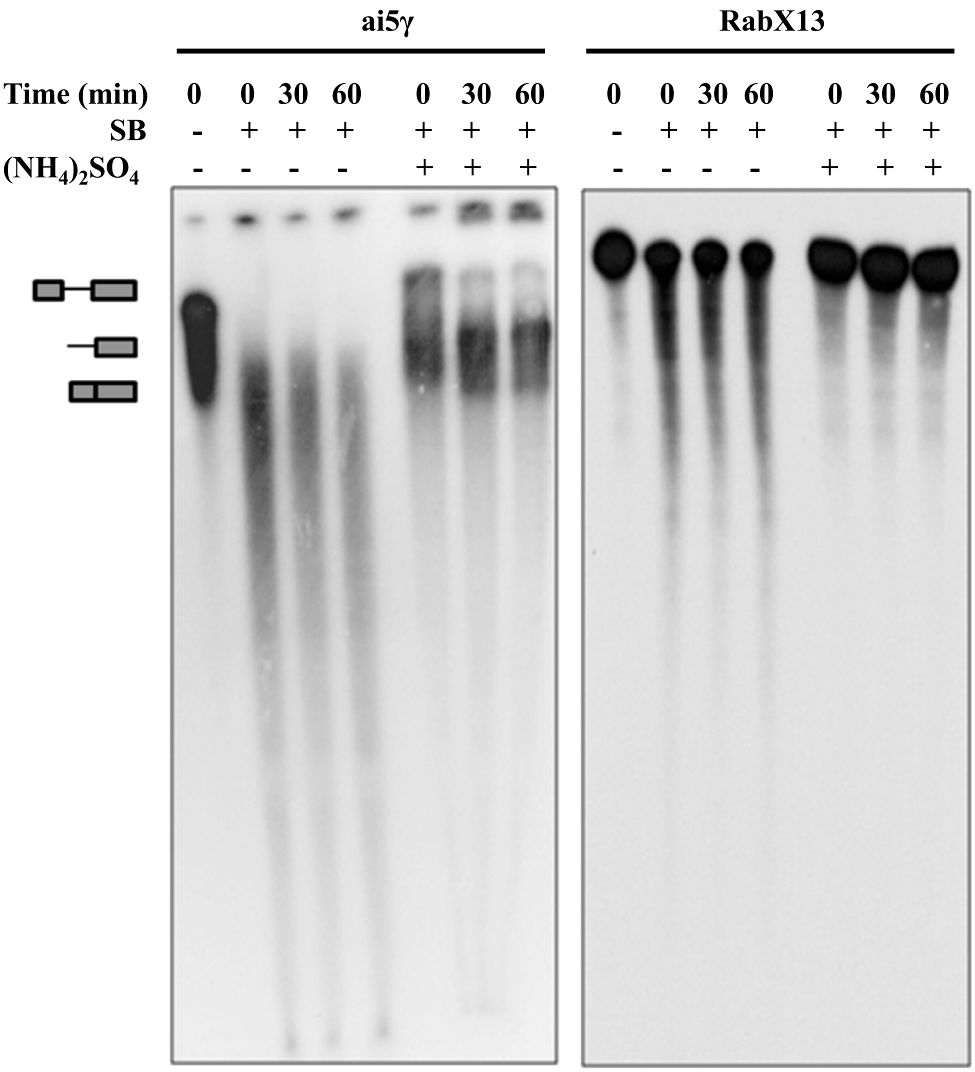

Figure S2. **RabX13 intron is not self-spliced**. ai5γ group II intron (left) and an intron-containing RabX13 (right) ^{32}P-labelled transcripts[1] were analyzed in self-splicing conditions[2]. Splicing products are shown to the left: intron-3' exon intermediate and the mRNA, as observed previously[2]. SB, splicing buffer in absence (-) or presence (+) of 0.5 M $(NH_4)_2SO_4$ which favors ai5γ intron self-splicing. No RabX13 splicing products were detected even with $(NH_4)_2SO_4$, suggesting that flicRNAs might not originate through this mechanism.

Unlike vertebrate ciRNAs, flicRNAs are not missing intronic regions resembling circularized group I and group II introns[3], which depend on sequence complementarity to attain high order structure driving their self-splicing reactions[4]. Amoeba introns may be structured as well, albeit not conserving group I and group II intron domains. Self-spliced intron circles are produced either by ligation of linear introns resulting from spliced exon reopening reactions[5], or by full-length intron circularization, consisting of a nucleophylic attack of the free 3'ss to the 5'ss in a 5' exon-intron byproduct resulting from downstream exon hydrolysis[6].

Methods for Figure S2

***In vitro* transcription and self-splicing reactions.**
Using gDNA as template, a 401 bp *RabX13* minigene comprising the whole intron and part of the flanking exons was amplified by PCR; the forward primer contained the T7 promoter. The RabX13 miningene and the ai5γ group II intron harbored in the pJD20 plasmid were transcribed *in vitro* as described (1) using [α-^{32}P]-UTP (Perkin Elmer) and the T7 RNA Polymerase (Ambion). The resulting RNA substrates were mixed in splicing buffer [40 mM Tris-HCl [pH7.5], 100 mM $MgCl_2$, 0.1% SDS] with or without 0.5 M $(NH_4)_2SO_4$ and incubated at 45°C (2). Reactions were stopped by addition of one volume of formamide buffer, and were immediately fractionated in denaturing 8M urea-polyacrylamide gels. Splicing products were visualized by autoradiography.

References
[1]Martinez-Contreras R, Galindo JM, Aguilar-Rojas A, Valdes J. 2003. Two exonic elements in the flanking constitutive exons control the alternative splicing of the alpha exon of the ZO-1 pre-mRNA. *Biochimica et biophysica acta* 1630: 71-83.
[2]Jarrell KA, Dietrich RC, Perlman PS. 1988. Group II intron domain 5 facilitates a trans-splicing reaction. Molecular and cellular biology 8:2361-2366; Murray HL, Mikheeva S, Coljee VW, Turczyk BM, Donahue WF, Bar-Shalom A, Jarrell KA. 2001. Excision of group II introns as circles. Molecular cell 8:201-211.
[3]Saldanha R, Mohr G, Belfort M, Lambowitz AM. 1993. Group I and group II introns. FASEB journal 7:15-24; Simon DM, Clarke NA, McNeil BA, Johnson I, Pantuso D, Dai L, Chai D, Zimmerly S. 2008. Group II introns in eubacteria and archaea: ORF-less introns and new varieties. RNA 14:1704-1713.
[4]Pyle AM. 2016. Group II Intron Self-Splicing. Annu Rev Biophys 45:183-205.
[5]Monat C, Quiroga C, Laroche-Johnston F, Cousineau B. 2015. The Ll.LtrB intron from Lactococcus lactis excises as circles in vivo: insights into the group II intron circularization pathway. RNA 21:1286-1293.
[6]Hedberg A, Johansen SD. 2013. Nuclear group I introns in self-splicing and beyond. Mob DNA 4:17.
